# Hospital Quality and Racial Differences in Outcomes After Genitourinary Cancer Surgery

**DOI:** 10.1002/cam4.70436

**Published:** 2024-12-03

**Authors:** Christopher P. Dall, Xiu Liu, Kassem S. Faraj, Arnav Srivastava, Samuel R. Kaufman, Nicholas Hartman, Vahakn B. Shahinian, Brent K. Hollenbeck

**Affiliations:** ^1^ Department of Urology Massachusetts General Hospital Boston Massachusetts USA; ^2^ Department of Urology Brigham and Women's Hospital Boston Massachusetts USA; ^3^ Division of Health Services Research, Department of Urology University of Michigan Ann Arbor Michigan USA; ^4^ Department of Biostatistics, School of Public Health University of Michigan Ann Arbor Michigan USA; ^5^ Division of Nephrology, Department of Internal Medicine University of Michigan Ann Arbor Michigan USA

**Keywords:** outcomes, quality, race

## Abstract

**Introduction and Objectives:**

Prior work has demonstrated racial disparities in surgical outcomes for solid organ cancers. We sought to assess the relationship between hospital quality and racial disparities in achievement of textbook outcomes among patients undergoing surgery for prostate, kidney, and bladder cancer.

**Methods:**

We used 100% national Medicare Provider Analysis and Review files from 2017 to 2020 to assess textbook outcomes in Patients undergoing bladder (i.e., radical cystectomy), kidney (i.e., radical or partial nephrectomy), and prostate (i.e., radical prostatectomy) surgery for genitourinary malignancies. Our exposure was hospital‐level quality, assessed by the predicted to expected ratio of achievement of textbook outcomes, agnostic to social and economic determinants of health. Our main outcome was achievement of textbook outcomes in White and Black patients. We defined the textbook outcome as the absence of in‐hospital mortality, mortality within 30 days of surgery, readmission within 30 days of discharge, a postoperative complication, and prolonged length of stay. The secondary outcome was percentage of Black and White patients treated at the highest quality hospitals.

**Results:**

As hospital quality increased, disparities in the receipt of textbook outcome for White and Black patients narrowed. For every 0.1 increment increase in the predicted to expected ratio of hospital quality, Black‐White disparities in the odds of achieving textbook outcomes decreased by 5.7% (interaction OR: 1.06; 95% CI 1.01–1.11 *p* = 0.026). Black patients were less likely to be treated at the highest quality hospitals compared to White patients (45.2% vs. 49.5% *p* = < 0.001%).

**Conclusions:**

Compared to White patients, Black patients had lower odds of textbook outcomes after surgery for prostate, kidney, and bladder cancer. The racial differences in achieving textbook outcomes were narrowed as hospital quality increased. Black patients were less likely than White patients to be treated at the highest‐quality hospitals. Our findings underscore the importance of improved access to high quality care among Black patients.

## Graphical abstract

Patients undergoing bladder, kidney, or prostate cancer surgery at major teaching hospitals have higher rates of textbook outcomes when compared to patients at nonteaching hospitals. While the volume‐outcome relationship explains differences in kidney cancer, it does not fully account for disparities in outcomes seen in bladder and prostate cancer surgeries.

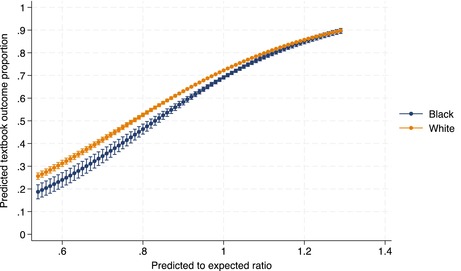

## 1 Introduction

Cancers of the prostate, kidney, and bladder are common, with more than 450,000 cases in the United States in 2023 [1]. Surgery is a common form of treatment for localized disease for these cancers but is associated with morbidity and, in some instances, mortality [2, 3]. Prior work has demonstrated that disadvantaged populations, including racial minorities, are at higher risk of worse outcomes after major cancer surgery [2, 4].

The extent to which hospital quality affects racial disparities in outcomes following surgery for cancers of the prostate, kidney, and bladder is uncertain, though evidence suggests that treatment location matters. In the community setting, for example, Black patients with muscle‐invasive bladder cancer had inferior survival relative to White patients after radical cystectomy [5]. Higher‐quality hospitals have the potential to mitigate racial differences in surgical outcomes through several mechanisms, including adherence to guideline‐concordant process measures perioperatively, earlier recognition and intervention of abnormal physiology postoperatively, and enhanced care coordination following discharge, among others [6, 7, 8, 9, 10, 11]. Alternatively, unconscious biases and/or other forms of structural racism may result in differences in healthcare delivery that maintain, or even exacerbate, disparities at high quality hospitals, particularly in circumstances in which the racial majority benefits disproportionately from interventions, as demonstrated in prior work [12, 13].

To better understand this issue, we performed a study to assess the relationship between hospital quality and racial disparities in outcomes after surgery for prostate, kidney, and bladder cancer.

## 2 Methods

We used a 100% sample of national Medicare Provider Analysis and Review files between January 1, 2017, and December 31, 2020, to identify patients, age 65 and older, undergoing elective major surgery for prostate (i.e., radical prostatectomy *n* = 61,385), kidney (i.e., partial or radical nephrectomy, *n* = 57,300), or bladder (i.e., radical cystectomy, *n* = 16,786) cancer. Patients were identified using *International Classification of Diseases*, tenth revision, diagnosis and procedure codes (Table S1). Patients discharged against medical advice (*n* = 70) and with missing covariates (*n* = 5433) were excluded. All patients were followed for at least 30 days postoperatively and thus discharges occurring in December 2020 (*n* = 2324) were excluded.

**FIGURE 1:**
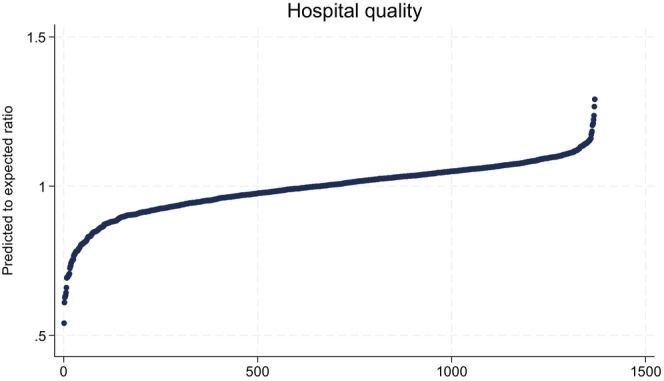
Hospital quality, measured by the predicted to expected ratio of achieving a textbook outcome, sorted from low to high.

The primary exposure for the study was hospital‐level quality as assessed by achieving textbook outcomes following the index procedure. A textbook outcome is a well‐established composite, binary measure used to define surgical quality by the absence of in‐hospital mortality, mortality within 30 days of the index surgery, readmission within 30 days of discharge, postoperative complications occurring during the index admission, and a prolonged length of stay [14, 15, 16, 17, 18, 19]. The last was defined by a hospitalization during the index admission longer than the 75th percentile for each surgery type in a calendar year. At the hospital‐level, race‐agnostic quality was defined by the predicted to expected ratio of textbook outcomes after the index operation, an approach used by the Centers for Medicare and Medicaid Services to account for varying patient characteristics and hospitals with low surgical volumes [20]. A predicted to expected ratio above one indicates that the hospital performs better than expected based on the national average and the hospital's patient case‐mix. Because of the focus on Black‐White disparities, this analysis necessarily was limited to hospitals caring for patients of both races. To promote reliability around the empirical estimate of hospital quality, the analysis was further limited to hospitals performing at least five procedures over the study period. These criteria resulted in excluding 604 hospitals and 6,040 patients. Predicted to expected ratios were estimated from a generalized linear mixed model including age, gender, surgical approach (conventional vs. minimally invasive), procedure type (prostatectomy, nephrectomy, and cystectomy), and comorbidity. Comorbidities were defined using 38 individual comorbidities defined by the Elixhauser comorbidity index [21]. Hospitals were incorporated in the models with random intercepts to account for clustering at the hospital level. The model estimating hospital quality was inclusive of patients from all racial/ethnic backgrounds and the ratios were not adjusted for race, dual‐eligibility, income, or any other social determinants of health. Confidence intervals for the predicted to expected ratios were established by bootstrapping the model over 1000 iterations. Figure 1 demonstrates the distribution of hospital quality measured by the predicted to expected ratio, which ranged from 0.54 to 1.29 with a median of 1.00.

### 2.1 Outcomes

The primary outcome for this study was achievement of a textbook outcome, measured at the patient level, among only White and Black patients undergoing major surgery for prostate, kidney, and bladder cancer. We excluded those from other racial backgrounds and those with missing racial identifiers. We hypothesized that any racial differences in textbook outcomes would diminish as hospital quality increased. As a secondary outcome, we measured the extent to which Black and White patients were managed at hospitals of varying levels of quality. For this purpose, hospitals were ranked based on their predicted to expected ratio measure of quality and sorted into tertiles, defining them as low (predicted to expected ratio of 0.54–0.97), medium (predicted to expected ratio of 0.97–1.04) and high (predicted to expected ratio of 1.04–1.29) quality. We hypothesized that Black patients would be disproportionately cared for at low quality hospitals.

### 2.2 Analysis

Differences between patients based on race were contrasted using *t*‐test and chi‐square tests for continuous and discrete data, respectively. Chi‐square tests were similarly used to assess differences in the probability of being treated at the highest quality hospitals. Multivariable logistic regression was used to model the relationship between achievement of a textbook outcome, patient race and hospital quality. An interaction term between patient race and hospital quality, measured at 0.1 increments in the predicted to expected ratio, was included in the model to estimate the probabilities of textbook outcomes in Black and White patients. The model was specified to estimate the probabilities of textbook outcomes for each surgery type at different levels of hospital quality and different race categories based on the average value of the covariates. Models were adjusted for patient age, gender, dual eligibility, surgical approach (conventional vs. minimally invasive), procedure type (prostatectomy, nephrectomy, and cystectomy), comorbidity, and dual eligibility for Medicare [21]. In addition, we adjusted for hospital bed number (< 100, 100–299, 300–499, or > 500 beds), ownership (private‐for‐profit, private‐for‐non‐profit, and government), and surgical volume (annual counts of procedures at a hospital sorted into tertiles).

All analyses were carried out using STATA software (version 18.0, StataCorpLLC). All tests were two‐sided, and the probability of type 1 error (*α*) was set at 0.05. The study protocol was approved by our institutional review board.

## 3 Results

Table 1 illustrates patient and hospital characteristics, stratified by race. Relative to White patients, Black patients were younger, were more likely to be female, and had more comorbidities. For example, the average age of Black and White patients undergoing surgery was 70.4 and 72.2, respectively (*p* < 0.001).

**TABLE 1:** Patient characteristics.

	White	Black	*p*
(% of cohort)			
Prostate	48,419 (88.0%)	6592 (12.0%)	
Kidney	45,678 (89.2%)	5519 (10.8%)	
Bladder	14,837 (94.4%)	873 (5.6%)	
Age (SD)	72.2 (5.4)	70.4 (4.8)	< 0.001
Gender (*n* male, % male)	10,134 (78.1%)	88,155 (80.9%)	< 0.001
Elixhauser (mean, SD)	2.0 (1.6)	2.4 (1.7)	< 0.001
Percentage dual eligible (*n*, %)	6024 (5.5%)	2484 (19.1%)	< 0.001
Bed number			< 0.001
< 100	3393 (3.2%)	300 (2.3%)	
100–299	29,544 (27.1%)	3064 (23.6%)	
300–499	29,228 (26.8%)	3485 (26.8%)	
≥ 500	46,769 (42.9%)	6135 (47.3%)	
Volume category			< 0.001
Low (< 116 procedures)	25,435 (23.3%)	3366 (25.9%)	
Average (116–260 procedures)	29,605 (27.2%)	3745 (28.9%)	
High (≥ 261 procedures)	53,894 (49.5%)	5873 (45.2%)	
Ownership type			< 0.001
Government	12,195 (11.2%)	1615 (12.4%)	
Private‐for profit	9862 (9.1%)	1272 (9.8%)	
Private‐for‐nonprofit	86,877 (79.8%)	10,097 (77.8%)	

Abbreviation: SD: standard deviation.

**FIGURE 2:**
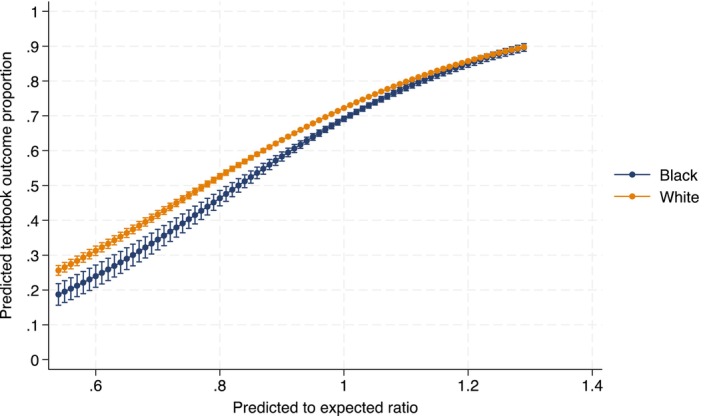
Predicted probability of achieving a textbook outcome after surgery for prostate, kidney, and bladder cancer by hospital quality. Probability of textbook outcome.

**FIGURE 3:**
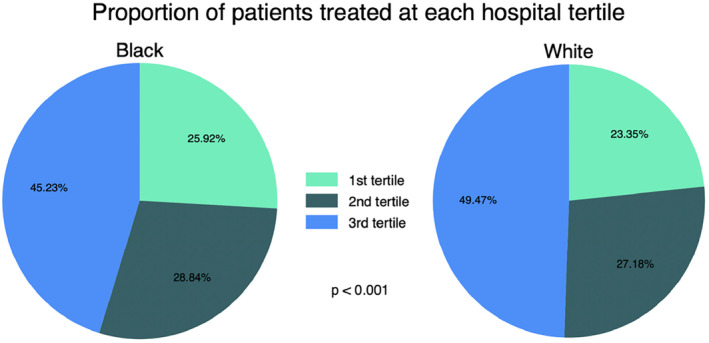
Percentage of patients, by race, treated within each hospital tertile.

Among 121,918 patients undergoing urologic cancer surgery at 1369 hospitals, textbook outcomes were achieved in 8916/12,984 (68.7%) Black patients and 80,003/108,934 (73.4%) White patients (*p* < 0.001). However, as shown in Figure 2, the interaction of race and quality was significant, indicating that the relationship between hospital quality and textbook outcomes differed by race. For every 0.1 unit increase in the predicted to expected ratio, Black‐White disparities in the odds of achieving in textbook outcomes (as measured by the odds ratio) decreased by 5.7% (interaction OR: 1.06; 95% CI 1.01–1.11 *p* = 0.026). This resulted in an attenuation of the gap in the predicted probability of textbook outcomes between White and Black patients. For example, at a hospital with a predicted to expected ratio at the 25th percentile (ratio = 0.95), rates of textbook outcomes between White and Black patients were 72.7% versus 68.9% for prostate surgery, 68.4% versus 64.3% for kidney surgery and 50.5% versus 45.8% for bladder surgery. However, at hospitals ranked in the 75th percentile (ratio = 1.05), more equitable percentages of textbook outcomes between White and Black patients were observed for prostate (80.5% vs. 78.4%), kidney (77.1% vs. 74.7%), and bladder (61.6% vs. 58.4%) surgery. Separate analyses stratified by surgery type yielded results similar in direction and magnitude (Table S2). Fewer Black patients were treated at highest quality (i.e., top tertile) compared to White patients (45.2% vs. 49.5%, *p* < 0.001) (Figure 3).

## 4 Discussion

Our analysis demonstrates that Black patients, on average, are less likely to achieve textbook outcomes after surgery for prostate, kidney, and bladder cancer compared to White patients. However, racial disparities in achieving textbook outcomes are attenuated for Black patients as hospital quality increases. Finally, Black patients are less likely than White patients to be treated at the highest quality hospitals.

Prior work evaluating factors associated with racial disparities in urologic cancer have produced conflicting results. Using the National Cancer Database, Black patients with bladder cancer were more likely to be diagnosed with locally advanced (OR: 1.49; *p* < 0.05) or metastatic (OR: 1.66; *p* < 0.05) disease and were less likely to receive treatment at both early (OR 0.68; *p* < 0.05) and late stages (OR: 0.65–0.79; *p* < 0.05) [22]. More broadly, disparities between White and Black Medicare beneficiaries increased in more affluent neighborhoods following general surgery procedures, suggesting that improvements in healthcare may preferentially affect racial majorities [13]. However, at least one study, which matched patients based on comorbidities, found no differences in mortality following general surgery procedures, instead concluding that the poorer health of Black patients was related to worse outcomes [23].

Our findings should be interpreted in the context of its limitations. First, our study uses Medicare data, which does not provide cancer‐specific disease characteristics that may confound results. However, we adjust for comorbidities and patient characteristics known to affect short‐term outcomes and we expect disease biology to have a limited impact over the time horizon (30‐days post‐discharge) assessed [24, 25, 26]. Additionally, as a cross‐sectional study, our findings do not imply causality. However, we control for differences in hospital characteristics. Further, our findings, in addition to those in other surgical fields, support the plausibility that hospital factors can mitigate or amplify racial disparities [27, 28, 29]. Finally, our data is limited to an older population that generally has access to healthcare coverage and thus may not be generalizable to the US population. In younger populations, racial differences in access to healthcare may be even more pronounced [30], suggesting that our findings may underestimate disparities [31].

Despite these limitations, this study has important implications that improve our understanding of racial disparities following major cancer surgery. It provides evidence that higher quality hospitals attenuate disparities between White and Black patients, lending support to importance of hospital selection. That increasing hospital quality is associated with narrowing of the gap in surgical outcomes may reflect more diverse staffing, improved access to care, or more universal adherence to guideline‐concordant care [32, 33, 34]. Our work, similar to prior data published on racial disparities in access to care, demonstrates that Black patients are less likely to be treated at hospitals with the best outcomes, which may further exacerbate inequities in outcomes [35]. Prior research has demonstrated that hospitals providing care for higher proportions of Black patients received fewer revenues for patient care compared to other hospitals, suggesting systemic economic disparities [36]. While our findings underscore the need to improve access of care to the highest quality hospitals among Black patients, our data suggest an imperative to also focus on quality improvements in low and medium quality hospitals, which may have the biggest impact on patient outcomes and racial disparities. Targeted investments in quality improvement programs at these underperforming hospitals may yield to process measures that reduce disparities and improve outcomes for both Black and White patients. Additionally, our data suggest that disparities among Black and White patients may be a benchmark by which overall hospital quality may be measured, providing an additional tool to gauge hospital quality.

In conclusion, higher‐quality hospitals mitigated racial disparities in achieving textbook outcomes after major urologic cancer surgery. Black patients were less likely to receive care at high quality hospitals than White patients. Further work should explore care processes and more granular hospital qualities that may underlie residual differences so as to eliminate disparities for all.

## Author Contributions

**Christopher P. Dall:** conceptualization (equal), formal analysis (equal), investigation (equal), methodology (equal), project administration (equal), visualization (equal), writing – original draft (equal), writing – review and editing (equal). **Xiu Liu:** formal analysis (equal), methodology (equal). **Kassem S. Faraj:** formal analysis (equal), writing – review and editing (equal). **Arnav Srivastava:** formal analysis (equal), writing – review and editing (equal). **Samuel R. Kaufman:** formal analysis (equal), investigation (equal), methodology (equal), supervision (equal), writing – review and editing (equal). **Nicholas Hartman:** validation (equal), writing – review and editing (equal). **Vahakn B. Shahinian:** conceptualization (equal), formal analysis (equal), investigation (equal), methodology (equal), project administration (equal), supervision (equal), writing – review and editing (equal). **Brent K. Hollenbeck:** conceptualization (equal), formal analysis (equal), investigation (equal), resources (equal), supervision (equal), writing – review and editing (equal).

## Ethics Statement

This study was approved by the Massachusetts General Brigham Institutional Review Board and was exempted from informed consent.

## Conflicts of Interest

The authors declare no conflicts of interest.

## Supporting information


**Table S1.** ICD 10 diagnosis and procedure codes used in analysis.

**Table S2.** Stratified analysis by surgery type.

## Data Availability

Medicare data is not available for public sharing.
