# Supplementary material for: Hospital Quality and Racial Differences in Outcomes After Genitourinary Cancer Surgery
Source: Cancer Med. 2024 Dec 3;13(23):e70436. doi: 10.1002/cam4.70436 (PMC11612663; doi:10.1002/cam4.70436)
Supplement: Supplementary file 1 — Table S1. ICD 10 diagnosis and procedure codes used in analysis. [file CAM4-13-e70436-s001.docx]

|  | ICD-10 Diagnosis Codes | ICD-10 Procedure Codes | |
| --- | --- | --- | --- |
|  |  | Open | MIS |
| Kidney surgery | \| C64 \| \| --- \| \| C64.1 \| \| C64.2 \| \| C64.9 \| \| D41.00 \| \| D41.01 \| \| D41.02 \| \| D41.9 \| \| C68.9 \| \| C68.8 \| \| C48.0 \| \| D30.00 \| \| D30.01 \| \| D30.02 \| | 0TB00ZZ  0TB10ZZ  0TT00ZZ  0TT20ZZ  0TT10ZZ | 0TB04ZZ  0TB14ZZ  0TT04ZZ  0TT24ZZ  0TT14ZZ |
| Bladder surgery | C67.x | 0TTB0ZZ | 0TTB4ZZ |
| Prostate Surgery | C61 | 0VB00ZZ  0VT00ZZ  0VT07ZZ | 0VB03ZZ  0VB04ZZ  0VT04ZZ  0VT08ZZ |

Supplementary Table 1: ICD 10 diagnosis and procedure codes used in analysis
