# Supplementary material for: Hospital Quality and Racial Differences in Outcomes After Genitourinary Cancer Surgery
Source: Cancer Med. 2024 Dec 3;13(23):e70436. doi: 10.1002/cam4.70436 (PMC11612663; doi:10.1002/cam4.70436)
Supplement: Supplementary file 2 — Table S2. Stratified analysis by surgery type. [file CAM4-13-e70436-s002.docx]

Supplemental Table 2: Stratified analysis by surgery type

| Surgery type | Estimate | 95% Confidence interval | p-value |
| --- | --- | --- | --- |
| Prostate | 1.85 | 0.93-3.67 | 0.080 |
| Kidney | 1.07 | 0.99-1.17 | 0.091 |
| Bladder | 1.12 | 0.97-1.30 | 0.128 |
